# Transcriptome profiling of claw muscle of the mud crab (*Scylla paramamosain*) at different fattening stages

**DOI:** 10.1371/journal.pone.0188067

**Published:** 2017-11-15

**Authors:** Qingling Jiang, Chenchang Bao, Ya’nan Yang, An Liu, Fang Liu, Huiyang Huang, Haihui Ye

**Affiliations:** 1 College of Ocean and Earth Sciences, Xiamen University, Xiamen, China; 2 Collaborative Innovation Center for Development and Utilization of Marine Biological Resources, Xiamen, China; Zhejiang University College of Life Sciences, CHINA

## Abstract

In crustaceans, muscle growth and development is complicated, and to date substantial knowledge gaps exist. In this study, the claw muscle, hepatopancreas and nervous tissue of the mud crab (*Scylla paramamosain*) were collected at three fattening stages for sequence by the Illumina sequencing. A total of 127.87 Gb clean data with no less than 3.94 Gb generated for each sample and the cycleQ30 percentages were more than 86.13% for all samples. De Bruijn assembly of these clean data produced 94,853 unigenes, thereinto, 50,059 unigenes were found in claw muscle. A total of 121 differentially expressed genes (DEGs) were revealed in claw muscle from the three fattening stages with a Padj value < 0.01, including 63 genes with annotation. Functional annotation and enrichment analysis showed that the DEGs clusters represented the predominant gene catalog with roles in biochemical processes (glycolysis, phosphorylation and regulation of transcription), molecular function (ATP binding, 6-phosphofructokinase activity, and sequence-specific DNA binding) and cellular component (6-phosphofructokinase complex, plasma membrane, and integral component of membrane). qRT-PCR was employed to further validate certain DEGs. Single nucleotide polymorphism (SNP) analysis obtained 159,322, 125,963 and 166,279 potential SNPs from the muscle transcriptome at stage B, stage C and stage D, respectively. In addition, there were sixteen neuropeptide transcripts being predicted in the claw muscle. The present study provides a comprehensive transcriptome of claw muscle of *S*. *paramamosain* during fattening, providing a basis for screening the functional genes that may affect muscle growth of *S*. *paramamosain*.

## Introduction

The mud crab *Scylla paramamosain* belonging to genus *Scylla*, is widely distributed along inshore region in the southeast China coasts and other Asian countries [1]. Due to the short growth cycle and the large market demand, the mud crab has high commercial value in farming [2]. The aquaculture of this species has emerged more than 100 years in China and more than 30 years in other Asian countries [1, 3]. The lower yields own to many factors, such as growth, nutrition, diseases, and administration patterns [4]. Therefore, research into mechanism underlying the growth and development is of obvious importance.

In crustaceans, the growth demands periodic shedding and replacement of the exoskeleton. Thereinto, the atrophy and restoration of muscle accompanied by each molt process was crucial. Muscle growth is accompanied with the increase in the number and length of muscle fibers, which were related to the molting [5]. Previous studies have shown that the muscle fibers grow in length immediately after molting in fully differentiated lobster [6]. The large myofibril splits in molting, and then grows in the cross-sectional area during the inter-molt [6]. In crabs, it is well known that the body weight increased significantly during the fattening process, nevertheless the molecular mechanism of muscle growth is unclear so far.

The transcriptomes of various tissues have been reported in *S*. *paramamosain*, such as hemolymph, gonads, cerebral ganglia, gill and mixture of the muscle, hepatopancreas, eyestalk [4, 7–9], while the transcriptome of muscle is still unclear. This paper reported the results of next-generation sequencing (Illumina sequencing) which pay attention to claw muscle growth during mud crab fattening. Analysis were performed against the sequencing results, including De Bruijn assembly of transcriptome sequences, functional annotation, coding sequence (CDS) prediction, single nucleotide polymorphism (SNP) discovery, genes expression analysis, differential expression analysis. Data obtained in this study facilitate the in-depth understanding of the changes occurring in muscle of *S*. *paramamosain* at transcriptomic and molecular level, and could contribute to studies on specific functional genes, the construction of genetic map and identification of molecular markers in this species.

## Materials and methods

### Sample collection

*S*. *paramamosain* (carapace width 7.5–8.4 cm, body weight 80–130 g) were obtained from a local aquafarm, Haicang District, Xiamen, China (24˚31’24.04”N; 118˚03’18.33”E). They were reared in tanks (temperature: 27 ± 2°C; salinity: 26 ± 1 ppm), and fed with the meat of the white Pacific shrimp *Litopenaeus vannamei* [10]. Crabs at three different fattening stages were sampled, according to the molt cycle of the swimming crab *Portunus trituberculatus* [11], they attribute to stage B (the first phase of the inter-molt period, the mud crab is very thin, body weight 80–90 g, called “empty” crab.), stage C (the dominant phase of the inter-molt period, body weight 105–120 g, the mud crab is full of meat.) and stage D (the fourth phase of the pre-molt period, body weight 125–130 g, namely double shell crab.). The crabs were dissected after anesthesia on ice for 30 minutes. The claw musculatures, nervous tissue (mixed tissues of cerebral ganglia, eyestalk, and thoracic ganglia) and hepatopancreas were collected, respectively. Three biological replicates were performed in per tissue at three stages, receiving a total of 27 samples. The study does not involve endangered or protected species.

### RNA extraction and cDNA library preparation

Total RNA were isolated with the Trizol Reagent (Invitrogen, USA) according to the manufacturer’s instructions. Then the extracted RNA was sequenced using the Illumina HiSeq 2500 by BMK (Beijing Co. Ltd). In short, mRNA poly (A) was separated using oligo (dT) beads. The second-strand cDNA fragments were synthesized using the purified fragments. After screening, the fragments were used for PCR amplification.

### Pre-processing and de bruijn assembly

The clean reads were guaranteed by clipping adapter, trimming low-quality reads and removing ambiguous bases. After pre-processing, they were assembled by Trinity using *de bruijn* algorithm [12]. All clean reads for *de bruijn* assembly were deposited in GenBank, National Centre for Biotechnology Information (NCBI) under the Accession No. PRJNA389966.

### Functional annotation

The unigenes were identified based on sequence similarity with known proteins and using a BLASTX (E≤1e-5) search against the Non-Redundant (NR), Swiss-Prot, Gene Ontology (GO), Clusters of Orthologous Groups (KOG). The KEGG Orthology of each unigene was analyzed using KOBAS 2.0 software [13]. Unigene annotation information was obtained using HMMER (E≤1e-10) search against the Pfam database with forecast of unigene amino acid sequence [14].

### Structural and expression analysis

Reliable potential CDS regions from the transcript sequences were identified by TransDecoder software, based on the length of open reading frame (ORF), the Log-likelihood Score, the amino acid sequence alignments with protein domain sequence in Pfam database and other information. Only reliable, STAR mapped reads were considered for SNPs detection. SNPs were called using GATK [15]. SNPs were qualified by continuous single nucleotide mismatch within 35 bp range no more than 3 and the value of standardized serialized SNP greater than 2.0. SNP markers were divided into homozygous- (only one allele) and heterozygous-SNP (two or more alleles). SNPs density in Unigenes was computed. All clean reads were aligned with Unigene database using Bowtie [16], followed performing the estimation of expression level combined RSEM [17]. The fragments per kilobase of exon model per million mapped reads [18] (FPKM) value indicates the corresponding unigene expression abundance.

### Identification and validation of differentially expressed genes

Correlation analysis was conducted with Pearson’s Correlation Coefficient (r) [19] to assess the relevance of biological replicates in the same condition. DESeq [20] was performed to analyze the differentially expressed genes (DEGs) between the samples in two conditions. Benjamini-Hochberg was employed to correct the significant value (p-value) for hypothesis test to reduce false positives. False discovery rate (FDR<0.01) and fold change (FC≥2) adopted as the key thresholds in difference expression genetic screening. Do hierarchical clusters to show differential expression patterns of gene sets under different experimental conditions. DEGs annotated in GO annotation database were enrichment analysis using the topGO software. COG statistical classification and KEGG annotation of DEGs were also performed. The significant enrichment of pathways was analyzed using the method of Fisher exact test.

Total RNA from claw muscle of specimens in three stages were extracted as described above. Approximately 2 μg RNA were used to the reverse transcription of cDNA. qRT-PCR was carry out in the 7500 Fast Real-Time PCR (Applied Biosystems) with 2×SYBR Select Master Mix (Applied Biosystems) to validate 10 DEGs (8 in the comparison of stage C and stage D, 2 in the comparison of stage B and stage C) expressed transcripts. Primers were designed using Primer5.0 Tool (PREMIER Biosoft International, Palo Alto, CA) with housekeeping gene 18s RNA as standard gene (Table 1). PCR reactions were performed under the following conditions: 95°C for 30 s, 40 cycles of 95°C for 5 s, 58°C for 30 s and 72°C for 30 s. Six biologic repetitions and three technical repetitions were performed in this study. The ultrapure water was the template in blank control. The 2^-ΔΔCt^ method was used to calculate the gene expression, and *Ct* values were the mean values of six biologic replicates [21]. The relative expression ratio was represented as mean±SD. All statistical analyses were performed using SPSS 18.0, including Duncan’s multiple range tests and the significance of differences analyzed using one-way ANOVA.

**Table 1:** The primers used in qRT-PCR.

Name	Sequence (5'→3')
*pfk-1-F*	ATCATTGGTGGATTCGAGGCTTA
*pfk-1-R*	CCTGCCAGTGTTGCCAAGTAG
*eno-F*	TGTTAGCAACCTACCAGACTCCAT
*eno-R*	GCCAGTTTCACTGATTCCACCTTA
*gapdh -F*	GATGTGTCCGTGGTTGACCTG
*gapdh -R*	CAATGACACGGTTGGAATAGCC
*Su(H)-F*	ACAGACAAGGCAGAATACCAGTTC
*Su(H)-R*	GAAATTCTCCCCGGTGAGCT
*hspb6-F*	GGAGGACGAGGCAAACTACAAGA
*hspb6-R*	GAGCAGTGATGGTAAGGACGC
*3-hao-F*	GATTATACTCACAACACCGTCC
*3-hao-R*	GTTCATATCTCCCTTTCTCATG
*mfs-F*	TTGTAGAGGTAGTTGCCGTAGATGG
*mfs-R*	AGTGGCTCACCCTCATCGTCTT
*ak-F*	CAACAATGGCTGACGCTGCT
*ak-R*	AGTTCTCAACACCGGACTGGATC
*cuti-F*	GAATGGCATCTACAAGAACGTGGTG
*cuti-R*	TCGGCGATTCGGATCTGCT
*mlc-F*	TCAAGAGTTGGATGAGATGCTGG
*mlc-R*	GTCGCAGTCAATGTTGCCCTC
*18S-F*	CAGACAAATCGCTCCACCAAC
*18S-R*	GACTCAACACGGGGAACCTCA

## Results

### Transcriptome sequencing and read assembly

**Fig 1:**
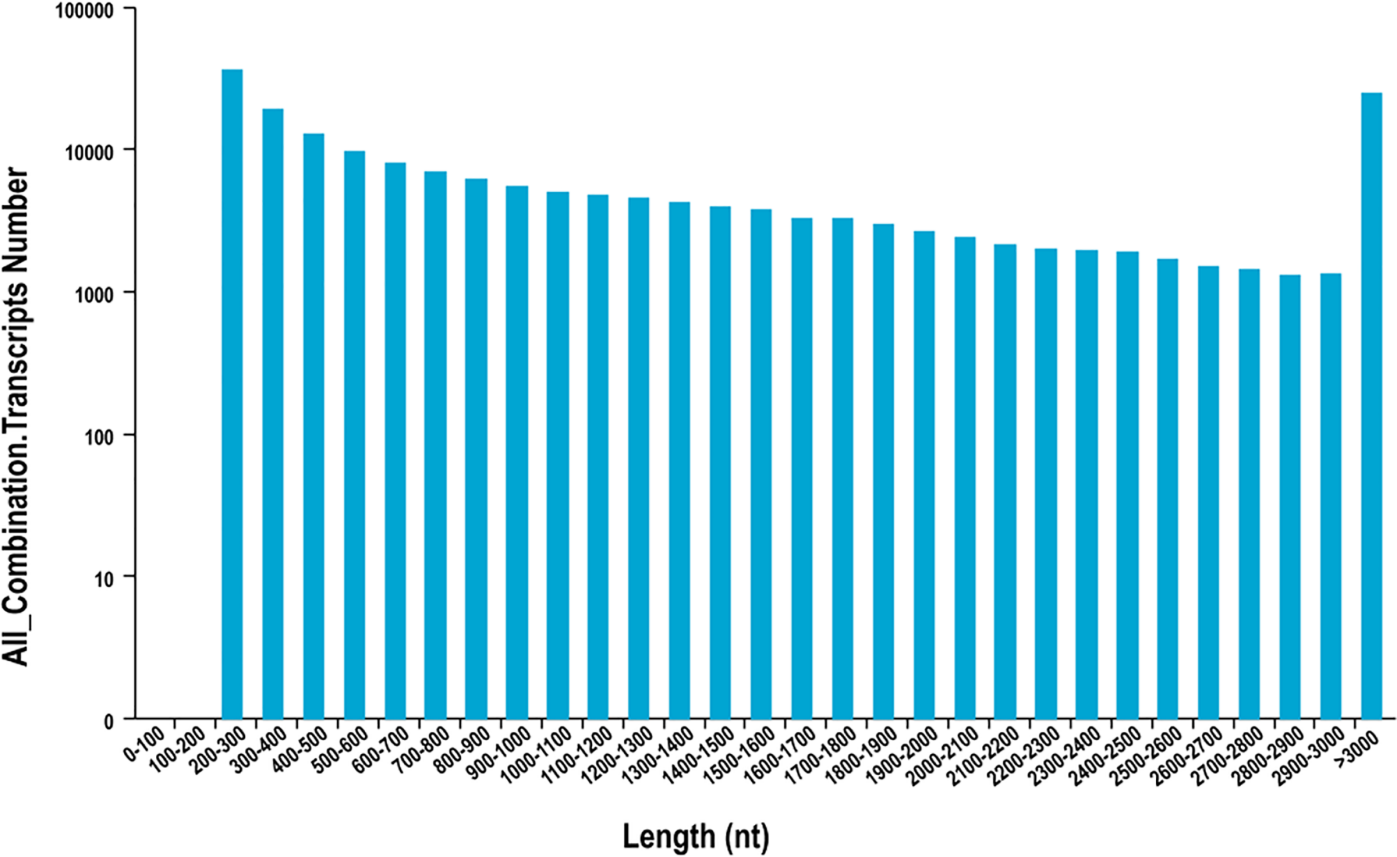
Length distribution summary of transcripts identified in *S*. *paramamosain* transcriptome data sets.

In this study, 27 cDNA libraries of *S*. *paramamosain* were sequenced using Illumina HiSeq 2500 platform. Clean sequencing reads and alignment statistics were showed in Table 2. The cycleQ30 percentage of all samples was more than 86.13%. The assembled transcripts (n = 183,760) had a total size of 291,529,594 bp, an average size of 1,586.47 bp and a N50 assembled transcripts with length of 3,031 bp. Nearly half of (54.56%) assembled transcripts were at the length range of 300–2000 nt (Fig 1).

**Table 2:** Summary of assembly statistics in *S*. *paramamosain*.

Samples		Clean sequencing reads			Alignment statistics		Assembled statistics			
		Total reads	Total bases (bp)	Percentage of GC content (%)	Mapped Reads	Mapped Ratio	Number of assembled transcripts	Total size of assembled transcripts (bp)	Number of assembled transcripts > 1K nt	Mean assembled transcripts size
**stage B**	H1	20,112,209	5,067,576,969	53.16%	15,584,072	77.49%	183,760	291,529,594	80,375	1,586.47
	H2	20,861,851	5,256,378,014	54.16%	16,429,685	78.75%				
	H3	18,655,143	4,700,470,289	51.38%	13,716,052	73.52%				
	M1	16,849,437	4,245,263,966	55.26%	13,273,186	78.78%				
	M2	16,742,590	4,218,417,849	51.62%	12,487,988	74.59%				
	M3	18,484,051	4,656,967,106	54.06%	14,370,747	77.75%				
	Y1	19,623,105	4,944,053,452	50.84%	15,563,551	79.31%				
	Y2	18,459,116	4,650,758,177	51.05%	14,275,073	77.33%				
	Y3	21,655,410	5,456,088,041	49.93%	17,013,947	78.57%				
**stage D**	H4	20,545,288	5,176,538,520	49.10%	16,426,588	79.95%				
	H5	17,907,718	4,511,999,917	49.20%	14,332,425	80.03%				
	H6	19,114,710	4,816,235,708	49.41%	15,239,510	79.73%				
	M4	18,637,615	4,695,854,468	52.79%	14,042,531	75.35%				
	M5	16,469,261	4,149,634,815	53.00%	12,736,660	77.34%				
	M6	16,937,402	4,267,442,962	54.92%	13,057,495	77.09%				
	Y4	19,271,980	4,855,825,679	57.30%	12,619,570	65.48%				
	Y5	20,509,246	5,167,672,936	53.67%	15,338,613	74.79%				
	Y6	23,266,613	5,862,342,086	53.76%	17,486,492	75.16%				
**stage C**	H7	23,294,003	5,869,102,468	50.08%	17,073,577	73.30%				
	H8	18,769,483	4,729,138,446	51.02%	14,332,336	76.36%				
	H9	17,573,006	4,427,451,799	50.37%	11,698,496	66.57%				
	M7	15,627,359	3,937,273,175	54.37%	12,298,736	78.70%				
	M8	16,849,106	4,245,259,370	54.04%	13,333,783	79.14%				
	M9	17,351,281	4,371,802,474	54.50%	13,739,437	79.18%				
	Y7	17,744,413	4,470,891,768	50.18%	13,908,009	78.38%				
	Y8	18,587,160	4,683,370,686	49.85%	14,497,722	78.00%				
	Y9	17,615,004	4,438,434,274	50.26%	13,946,640	79.17%				

Note: H1-3, H4-6, H7-9: the biological replicates of hepatopancreas at stage B, stage D, stage C; Y1-3, Y4-6, Y7-9: the biological replicates of nervous tissue at stage B, stage D, stage C; M1-3, M4-6, M7-9: the biological replicates of claw muscle at stage B, stage D, stage C.

### Functional annotation

**Fig 2:**
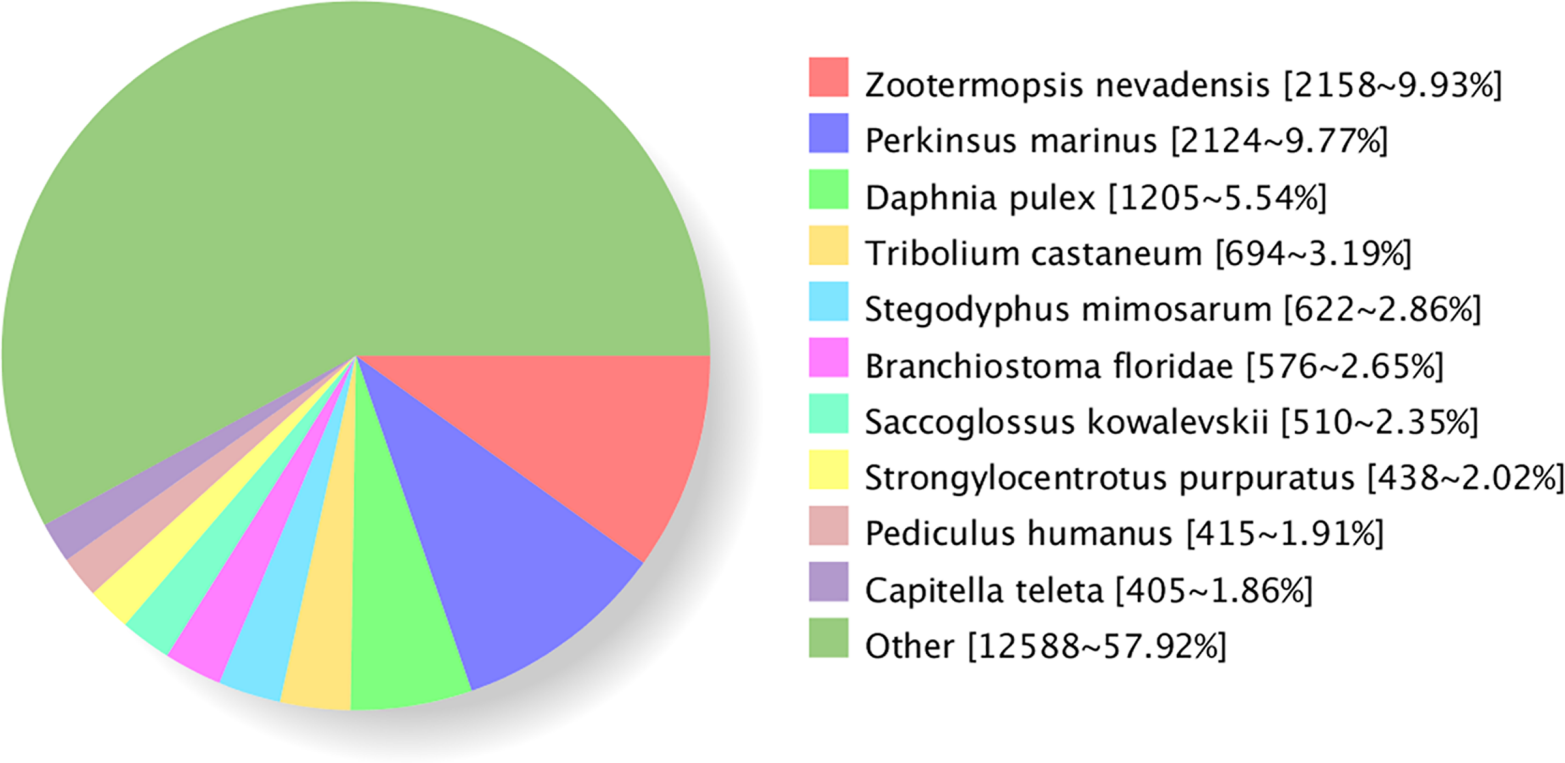
Nr homologous species distribution. The number of aligned genes and the percentage of which in all annotated genes were recorded in brackets.

In this study, 23,787 transcripts mapped back to the protein database. S1 and S2 Tables show the unigenes and the annotation information. Arthropods account for the largest proportion in homology analysis of *S*. *paramamosain* transcriptome. The top three organism were Nevada termite *Zootermopsis nevadensis* (9.93%), Alveolate *Perkinsus marinus* (9.77%) and Water flea *Daphnia pulex* (5.54%) (Fig 2). Table 3 shows the unigenes with highest quality annotations. In addition, 16 transcripts encoding neuropeptide precursors (13 complete and 3 partial) were identified from the transcriptome data (Table 4). The deduced neuropeptides include B-type allatostatin (AST-B), short Neuropeptide F (sNPF), neuroparsin (NP), crustacean hyperglycemic hormone (CHH), orcokinin, diuretic hormone 31 (DH31), tachykinin, myosuppressin, bursicon hormone alpha subunit, putative insulin-like protein growth factor binding protein and insulin-like androgenic gland factor.

**Table 3:** Top 20 annotations of *S*. *paramamosain* transcriptome with the highest bit score.

Description	Accession ID	Organism Scientific name (common name)	Alignment length (amino acids)	E value	Bit Score	Type
Dynein heavy chain, cytoplasmic	KDR21358.1	*Zootermopsis nevadensis* (Dampwood termite)	3,661	0	7,494	Full length
Ryanodine receptor	AGH68757.1	*Ostrinia furnacalis* (Asian corn borer)	3,178	0	6,187	Partial
Putative low-density lipoprotein receptor	JAB58180.1	*Corethrella appendiculata*	2,748	0	5,489	Partial
Cj-cadherin	BAD91056.1	*Caridina multidentata*	2,669	0	5,437	Full length
Projectin	BAC66140.1	*Procambarus clarkii* (Red swamp crayfish)	2,579	0	5,376	Partial
Uncharacterized protein	NV12534-PA	*Nasonia vitripennis* (Parasitic wasp)	2,168	0	4,564	Partial
Pre-mRNA-processing-splicing factor, putative	EEB14455.1	*Pediculus humanus* subsp. corporis (Body louse)	2,147	0	4,354	Full length
Uncharacterized protein	SMAR010770-PA	*Strigamia maritima* (European centipede)	2,194	0	4,335	Partial
Projectin	BAC66140.1	*Procambarus clarkii* (Red swamp crayfish)	2,184	0	4,221	Partial
Uncharacterized protein	SMAR006389-PA	*Strigamia maritima* (European centipede)	2,399	0	4,118	Partial
Spectrin alpha chain	KDR23504.1	*Zootermopsis nevadensis* (Dampwood termite)	2,010	0	4,052	Full length
target of rapamycin	AHX84170.1	*Fenneropenaeus chinensis*	2,105	0	4,009	Full length
microtubule-actin cross-linking factor 1-like	XP_008560478.1	*Microplitis demolitor*	2,106	0	3,903	Full length
spectrin beta chain, non-erythrocytic 2 isoform X3	XP_011150755.1	*Harpegnathos saltator*	2,057	0	3,890	Full length
neurofibromin	XP_008200683.1	*Tribolium castaneum*	1,924	0	3,821	Full length
E3 ubiquitin-protein ligase HERC2-like	XP_005096225.1	*Aplysia californica*	2,274	0	3,759	Full length
fat-like cadherin-related tumor suppressor homolog	XP_003491311.1	*Bombus impatiens*	2,096	0	3,674	Partial
myosin-VIIa-like isoform X1	XP_006621154.1	*Apis dorsata*	1,731	0	3,569	Full length
Laminin subunit alpha	KDR13939.1	*Zootermopsis nevadensis*	1,761	0	3,521	Partial
I-connectin	BAB64297.1	*Procambarus clarkii*	1,698	0	3,422	Full length

**Table 4:** Putative neuropeptide precursors in the muscle transcriptome of *S*. *paramamosain*.

Peptide families	Accession Num.	Size (bp)	Size (aa)	Best Blastx Match			
				Species name	E-value	Ident	Accession Num.
B-type allatostatin	K7RY75	1754	314	*Pandalopsis japonica*	0.0	100%	ALQ28584.1
short Neuropeptide F	U5EU21	1533	126	*S*. *paramamosain*	5e-89	100%	ALQ28574.1
Neuroparsin1	A0A023PY98	1782	101	*S*. *paramamosain*	5e-66	100%	ALQ28570.1
Neuroparsin2	W5S2B1	438	106	*S*. *paramamosain*	2e-71	100%	ALQ28588.1
Neuroparsin3	PF07327.6	794	97	*S*. *paramamosain*	2e-65	100%	ALQ28589.1
Neuroparsin4	W5S2B1	2399	102	*Metapenaeus ensis*	2e-67	100%	ALQ28571.1
CHH1	F2YLB0	1117	127	*S*. *paramamosain*	5e-91	99%	ALQ28582.1
CHH2	H9ZJK3	1847	140	*S*. *paramamosain*	2e-99	100%	ALQ28572.1
Orcokinin1	E9FTU8	1421	121	*S*. *paramamosain*	7E-83	100%	ALQ28595.1
Diuretic hormone 31	D2IJD5	1399	146	*S*. *paramamosain*	3e-100	100%	ALQ28573.1
Tachykinin	Q767J5	756	222	*S*. *paramamosain*	7e-156	99%	ALQ28591.1
Myosuppressin	B5BP38	803	100	*S*. *paramamosain*	4e-68	100%	ALQ28580.1
bursicon hormone alpha subunit	C3S7D8	1419	73	*Callinectes sapidus*	9e-45	99%	ACG50067.1
putative insulin-like protein growth factor binding protein	E4VP27	718	96	*Tityus obscurus*	8e-06	56%	JAT91097.1
insulin-like androgenic gland factor	A0A075INW9	879	68	*S*. *paramamosain*	6e-43	99%	AFY09903.1

**Fig 3:**
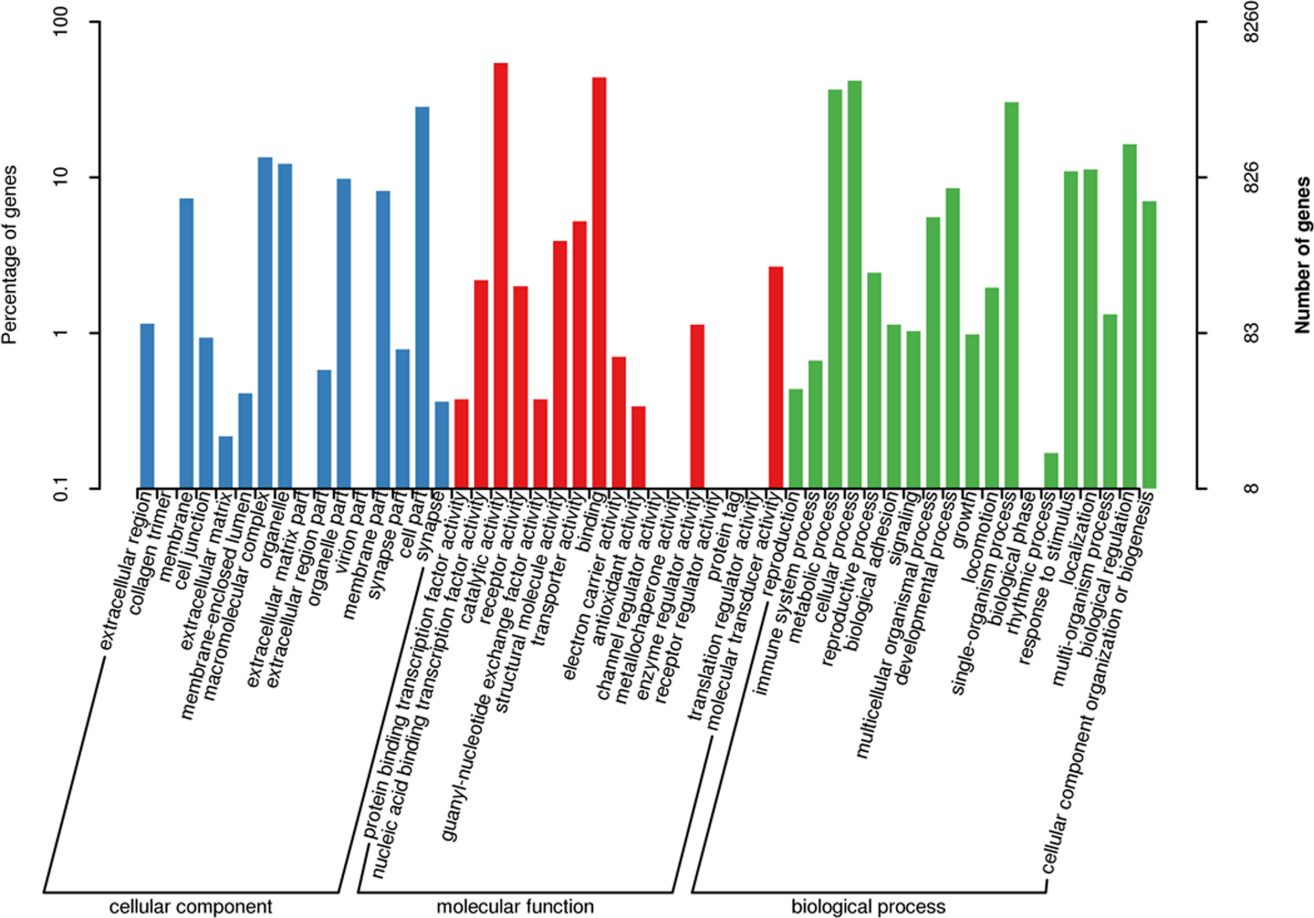
The classification of unigenes in three GO categories in *S*. *paramamosain*. The x-axis indicated GO process; the y-axis on the left side indicated the percentage of the unigenes of this process in all genes; the y-axis on the right side indicated the number of unigenes in the process.

**Fig 4:**
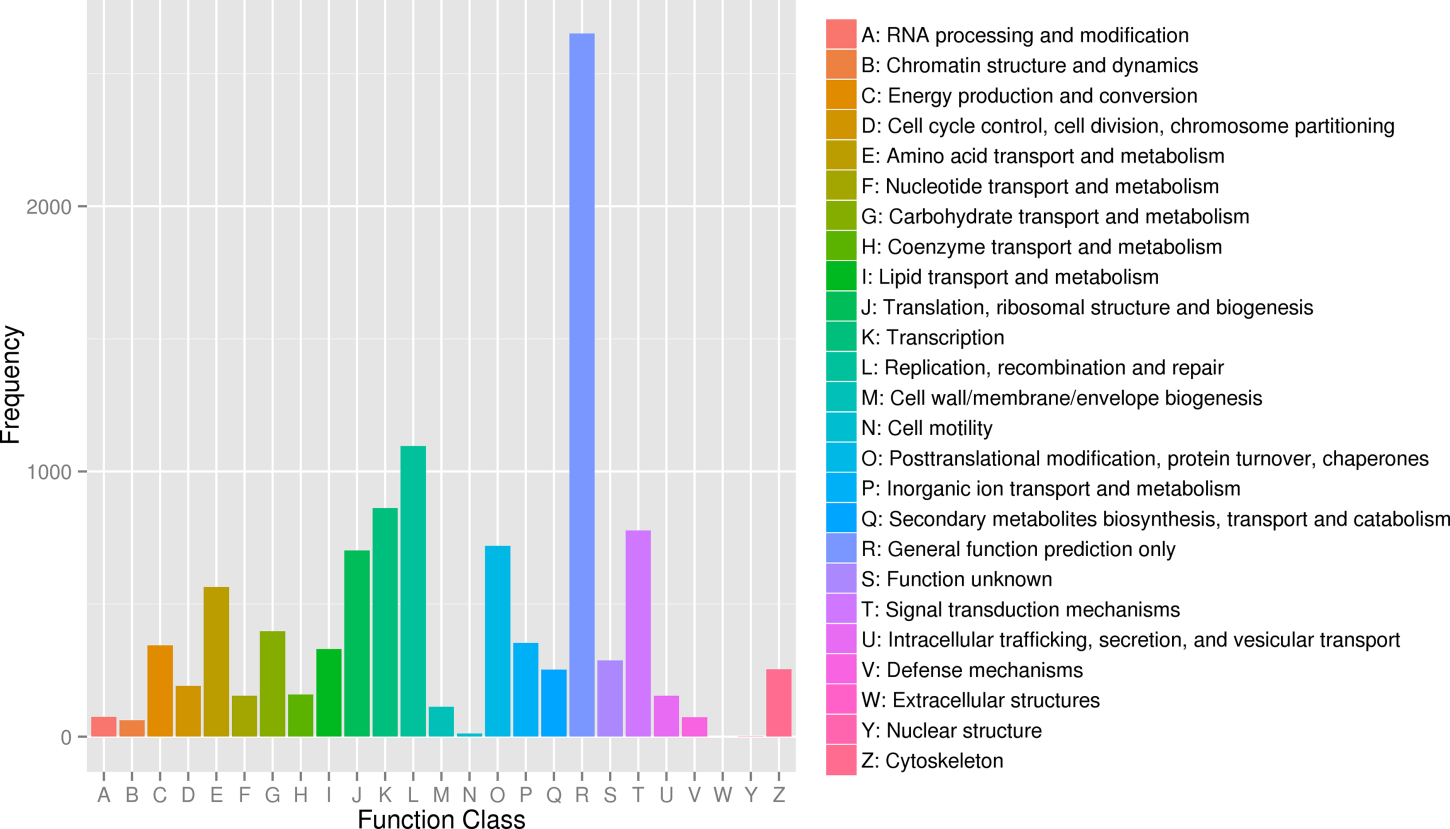
The COG function classification in *S*. *paramamosain*. The x-axis indicated COG function; the y-axis indicated the number of unigenes.

The analysis of GO terms showed that the 27,917 unigenes were in the GO domains (Fig 3). In biological domain, 19 terms contained 11,924 unigenes. The top three terms were metabolic process (285 unigenes), oxidation-reduction process (223 unigenes) and translation (214 unigenes). In cellular component domain, 4,596 unigenes were found in 16 terms. There were 429 unigenes involved in nucleus, followed by 383 unigenes in integral component of membrane and 376 unigenes in membrane. In molecular function domain, a total of 11,397 unigenes were distributed in 17 terms. The ATP binding (563 unigenes) and catalytic activity (459 unigenes) were top two terms. COG analysis showed that 7,742 transcripts were assigned to 25 COG terms, the top three COG terms of which were general function prediction (2,651 unigenes), replication, recombination and repair (1,096 unigenes) as well as transcription (862 unigenes) (Fig 4). In addition, 10,495 unigenes were classified into 220 KEGG pathways, of which 279 unigenes were in Protein processing in endoplasmic reticulum (ko: 04141), followed by 273 unigenes in oxidative Spliceosome (ko: 03040) and 270 unigenes in Purine metabolism (ko: 00230).

### Structural and expression analysis

A total of 56,328 CDS were predicted from 94,853 unigenes. CDS was not detected at sequence size 0–100 (bp). The top 3 levels were at sequence size 100–200 (bp), 200–300 (bp) and 300–400 (bp). As length increases, the number decreases. Therefore, the number of glimmer cds dropped significantly at sequence size 2700–2800 (bp), 2800–2900 (bp), 2900–3000 (bp). The length of CDS is shown in S1 Fig.

**Fig 5:**
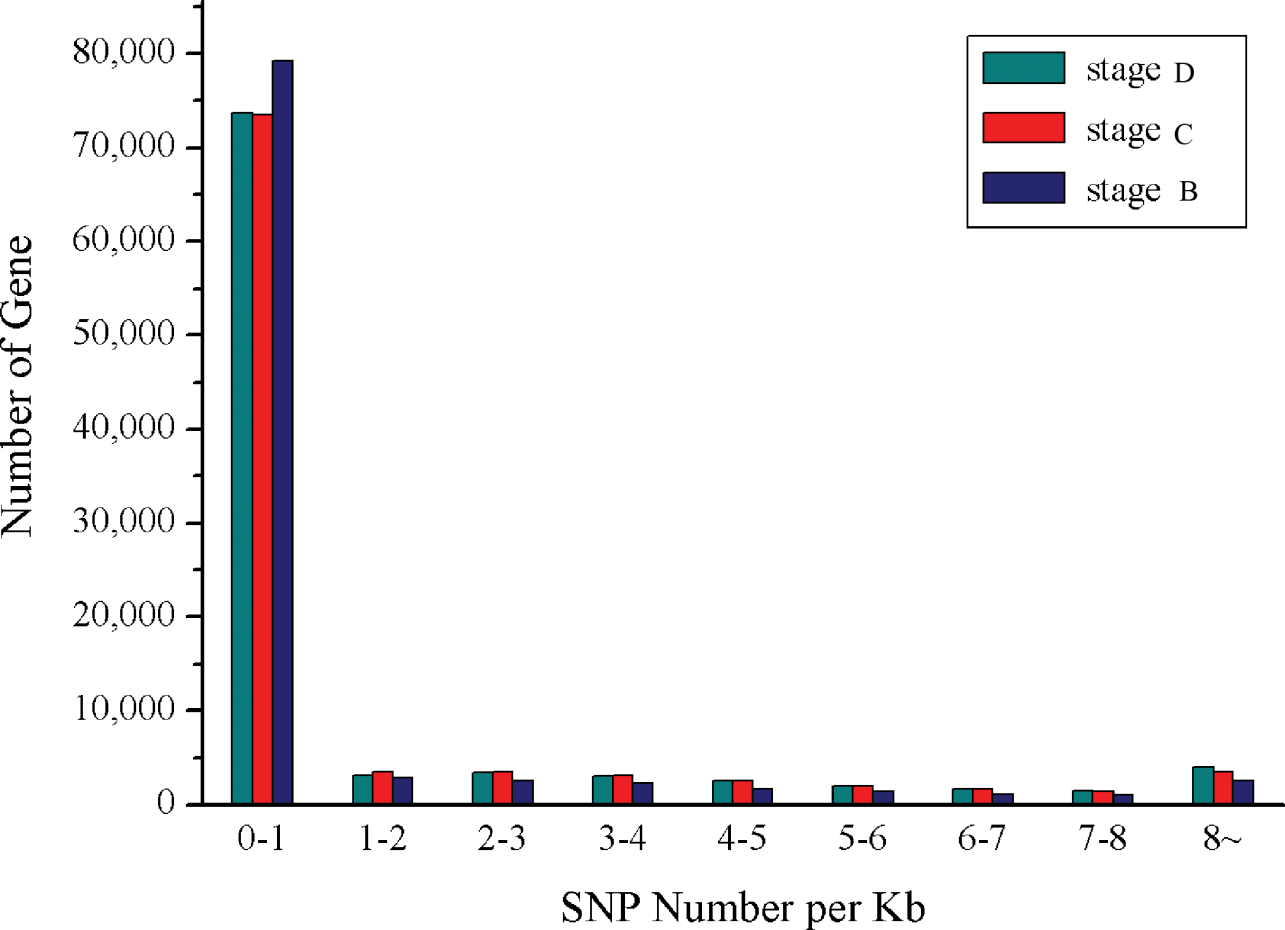
SNP density in *S*. *paramamosain*. The x-axis indicated SNP number per Kb; the y-axis indicated the number of unigene.

A total number of 159,322, 125,963 and 166,279 potential SNPs were obtained from stage B, stage C and stage D, including 143,546, 111,625 and 144,943 homoSNP, respectively (Table 5). The distribution of SNP in unigene is showed in Fig 5. SNP-free unigenes accounted for the largest proportion, followed by the unigenes with 0–1 SNP per Kb. As the density of SNPs increases in unigene, the number of unigene decreases. In addition, the correlation analysis has been performed between SNP and 121 DEGs of claw muscle transcriptomes (S5 Table). A total of 177 SNPs were found in 31 DEGs, thereinto, 105 SNPs in 12 annotated genes.

**Table 5:** Statistics of SNPs in *S*. *paramamosain*.

Samples	HomoSNP	HeteSNP	AllSNP
stage B	143,546	15,776	159,322
stage C	111,625	14,338	125,963
stage D	144,943	21,336	166,279

Note: HomoSNP: number of Homozygous SNP; HeteSNP: number of Hybrid SNP; All SNP: all number of SNP.

**Fig 6:**
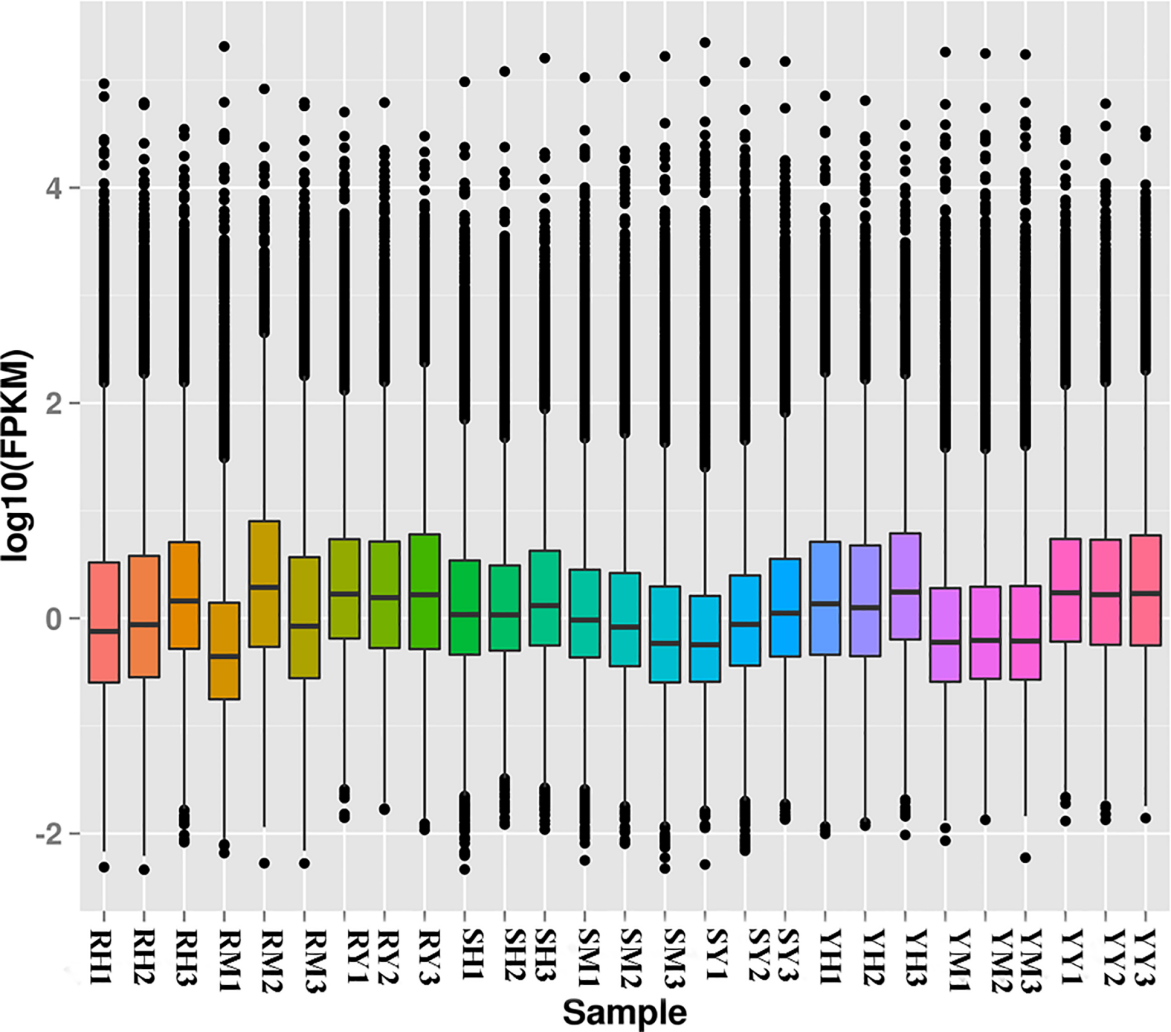
FPKM box chart in *S*. *paramamosain*. The x-axis indicated the samples; the y-axis indicated the logarithm of FPKM. The figure shows the overall level and the degree of dispersion of gene expression. The horizontal line in the box represents the expression level of 50% genes in the corresponding sample; black spots represent discrete genes, above the box for high expression, below the box for the low expression.

In this study, FPKM value indicates the unigene expression abundance (Fig 6). The results showed that the biologically repeated samples of nervous tissue behaved the highest consistency with the whole of three stages. In addition, the nervous tissue has the highest overall gene expression level, followed by hepatopancreas and muscle, while the dispersion degree of gene expression was opposite. As far as muscle sample, the stage D showed the highest overall gene expression level when compared with stage B and C. However, the reproducibility of muscle samples it is best at stage C. The overall genes expression level of hepatopancreas was slightly large at stage C.

### DEGs in the muscle transcriptome

**Fig 7:**
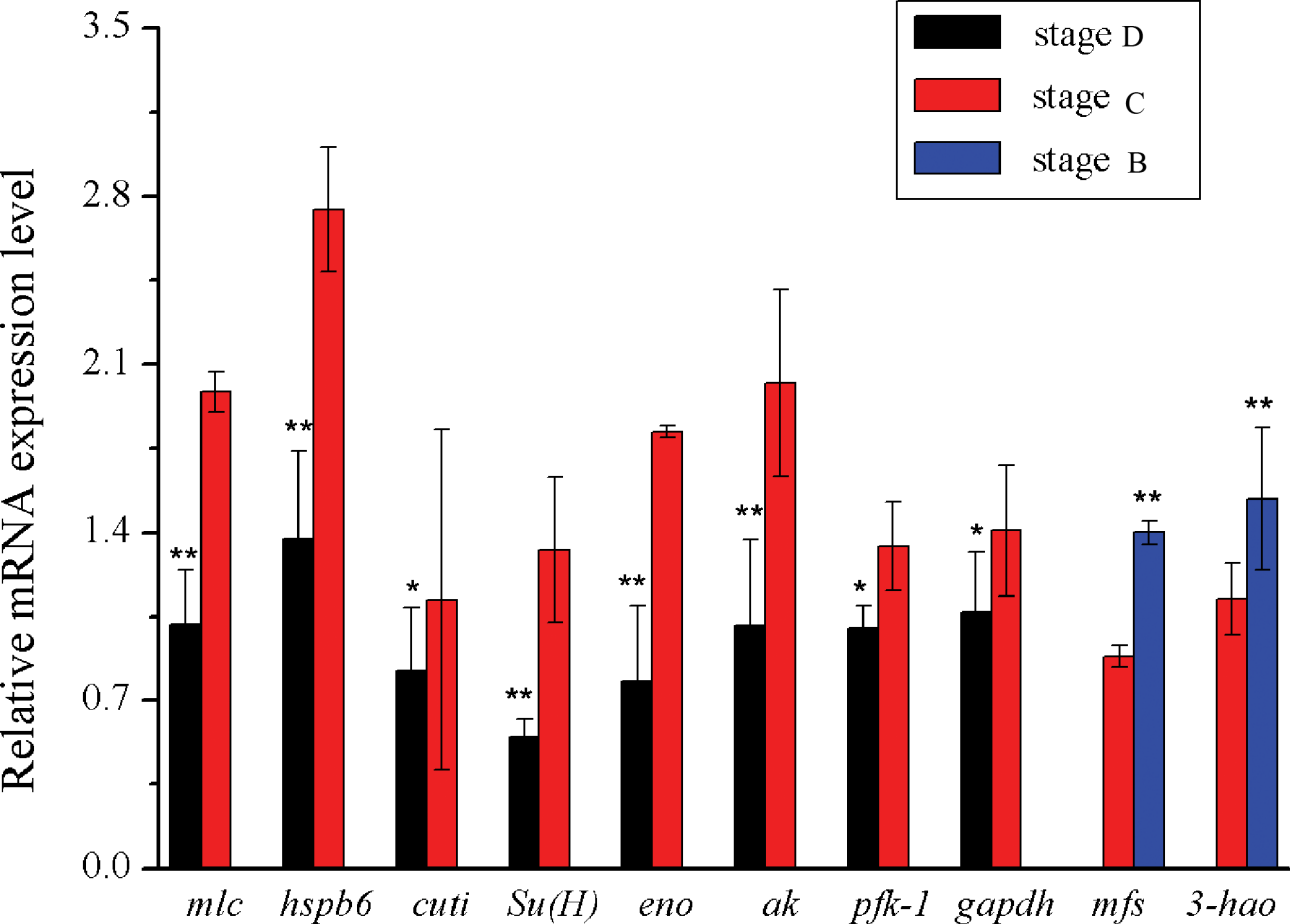
The DEGs of muscle of *S*. *paramamosain* at different stages. *pfk-1*: Phosphofructokinase-1; *eno*: Enolase; *gapdh*: Glyceraldehyde-3-phosphatede hydrogenase; *Su(H)*: Suppressor of Hairless; *hspb6*: alpha crystallin family; *3-hao*: 3-hydroxyanthranilic acid dioxygenase; *mfs*: Major Facilitator Superfamily; *ak*: Arginine kinase; *cuti*: arthrodial cuticle protein; *mlc*: myosin light chain; 18s RNA was used as reference gene. Values denoted by different numbers of asterisks were significantly different when compared by ANOVA (“**” p < 0.01; “*” p < 0.05).

The analysis of muscle transcriptome data revealed 121 DEGs at different stages, 63 (52.07%) of which were annotated successfully (Table 6). In the comparison of stage D and stage B, 2 genes (50% annotated, 50% unannotated) were identified and up-regulated at stage B. In the comparison of stage C and stage B, 23 genes (73.91% annotated, 26.09% unannotated) were identified, including 21 up-regulated genes and 2 down-regulated genes. In the comparison of stage C and stage D, 99 genes (46.46% annotated, 53.54% unannotated) were identified, including 29 up-regulated genes and 70 down-regulated genes. Moreover, 10 DEGs were validated by qRT-PCR, including arthrodial cuticle protein, phosphofructokinase-1 (PFK-1), enolase, arginine kinase (AK), suppressor of hairless [Su (H)], alpha crystallin family (HSPB6), glyceraldehyde-3-phosphate dehydrogenase (GAPDH) and myosin light chain (MLC) in the comparison of stage C and stage D, and major facilitator superfamily, 3-hydroxyanthranilic acid dioxygenase in the comparison of stage B and stage C. The results show that 3 genes (GAPDH, PFK-1, and arthrodial cuticle protein) only expressed differentially with *P* < 0.05, while the other seven showed consistent with the sequencing results (*P* < 0.01) (Fig 7).

**Table 6:** Statistics of DEGs in the muscle transcriptome in *S*. *paramamosain*.

		All genes			Significant DEGs (Padj < 0.01)	
Cond. 1	Cond.2	Total	Up- regulation (Cond.2> Cond.1)	Down- regulation (Cond.2< Cond.1)	Up- regulation (Cond.2> Cond.1)	Down- regulation (Cond.2< Cond.1)
stage D	stage B	13,323	5,907	7,416	2	0
stage C	stage B	10,171	5,204	4,967	21	2
stage C	stage D	11,153	5,984	5,169	29	70

Note: Cond.: condition.

### Functional analysis of DEGs of muscle

**Fig 8:**
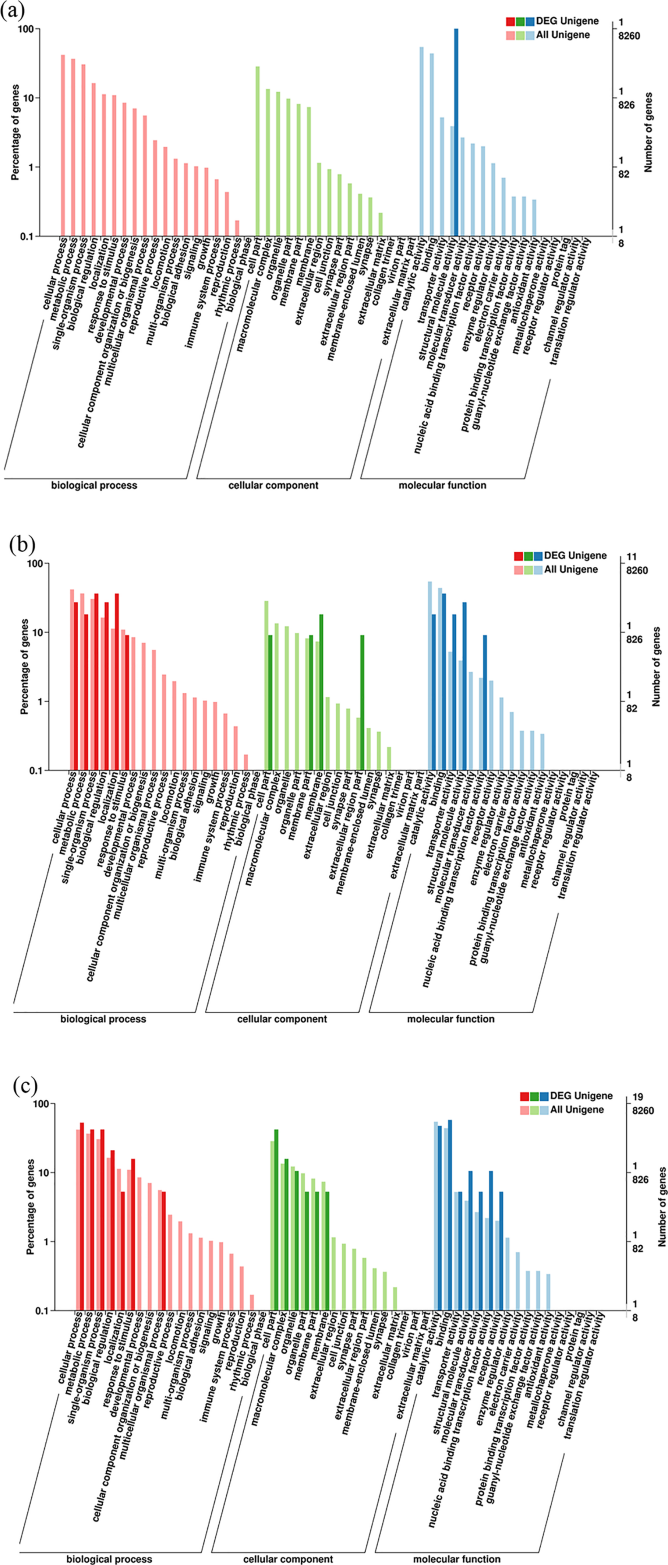
The GO categories of DEGs of muscle. (a) The GO categories of the DEGs in stage D specimens and stage B specimens. The x-axis indicated GO process; the y-axis on the left side indicated the percentage of the DEGs; the y-axis on the right side indicated the number of the DEGs (up) and all unigenes (down) in the process; (b) The GO categories of the DEGs in stage C specimens and stage B specimens; (c) The GO categories of the DEGs in stage D specimens and stage C specimens.

GO terms of DEGs were analyzed. In the comparison of stage D and stage B, the gene was annotated as calcified cuticle protein like gene and classified into structural molecule activity term (GO: 0042302) in molecular function domain (Fig 8A). In comparison of stage B and C, the DEGs were assigned to 15 terms, of which 6 genes in biological process, 3 genes in cellular component and 9 genes in molecular function. “single-organism process”, “membrane” and “binding” were the top terms in the three GO domains with 4, 2 and 4 genes, respectively (Fig 8B). In comparison of stage D and stage C, 46 genes were GO-categorized into biological process (13 genes), cellular component (8 genes) and molecular function (17 genes). The “cellular process” (10 genes), “cell part” (8 genes) and “binding and catalytic activity terms” (11 genes) were the top terms in the biological process domain, cellular component and molecular function process (Fig 8C). Table 7 shows the GO terms associated with significant DEGs.

**Table 7:** The GO terms with significant DEGs of muscle of *S*. *paramamosain*.

GO ID	GO term	All genes	DEGs	DEG IDs
**stage B VS stage C**				
**Biological_process**				
GO:0019363	pyridine nucleotide biosynthetic process	5	1	c51072.graph_c0
GO:0006355	regulation of transcription, DNA-templated	175	1	c40124.graph_c0
GO:0046427	positive regulation of JAK-STAT cascade	5	1	c2761.graph_c0
GO:0008039	synaptic target recognition	6	1	c51381.graph_c0
GO:0044765	single-organism transport	54	1	c41045.graph_c0
GO:0015918	sterol transport	1	1	c51381.graph_c0
GO:0055085	transmembrane transport	1	81	c5211.graph_c0
GO:0061057	peptidoglycan recognition protein signaling pathway	1	2	c51381.graph_c0
GO:0006810	Transport	1	184	c2761.graph_c0
GO:0055092	sterol homeostasis	1	1	c51381.graph_c0
GO:0019752	carboxylic acid metabolic process	1	6	c51072.graph_c0
GO:0045456	ecdysteroid biosynthetic process	1	2	c51381.graph_c0
**Molecular_function**				
GO:0070891	lipoteichoic acid binding	1	1	c51381.graph_c0
GO:0016491	oxidoreductase activity	1	206	c51072.graph_c0
GO:0042834	peptidoglycan binding	1	1	c51381.graph_c0
GO:0016812	hydrolase activity, acting on carbon-nitrogen (but not peptide) bonds, in cyclic amides	1	4	c34326.graph_c0
GO:0030882	lipid antigen binding	1	1	c51381.graph_c0
GO:0042302	structural constituent of cuticle	3	52	c53503.graph_c0;c52677.graph_c0;c54621.graph_c0
GO:0046914	transition metal ion binding	1	12	c34326.graph_c0
GO:0022891	substrate-specific transmembrane transporter activity	1	7	c2761.graph_c0
GO:0005215	transporter activity	1	44	c5211.graph_c0
GO:0043565	sequence-specific DNA binding	1	107	c40124.graph_c0
GO:0046872	metal ion binding	1	410	c51072.graph_c0
GO:0008270	zinc ion binding	1	214	c40124.graph_c0
GO:0003700	sequence-specific DNA binding transcription factor activity	1	135	c40124.graph_c0
GO:0001530	lipopolysaccharide binding	1	1	c51381.graph_c0
**Cellular_component**				
GO:0016021	Cellular Component: integral component of membrane	1	383	c5211.graph_c0
GO:0005886	Cellular Component: plasma membrane	1	132	c5211.graph_c0
GO:0016020	Cellular Component: membrane	1	376	c27537.graph_c0
GO:0005615	Cellular Component: extracellular space	1	25	c51381.graph_c0
**stage B VS stage D**				
**Molecular_function**				
GO:0042302	structural constituent of cuticle	1	52	c39692.graph_c0
**stage C VS stage D**				
**Biological_process**				
GO:0045454	cell redox homeostasis	1	20	c38096.graph_c1
GO:0006497	protein lipidation	1	1	c63835.graph_c0
GO:0005975	carbohydrate metabolic process	1	56	c46569.graph_c0
GO:0006355	regulation of transcription, DNA-templated	2	175	c49393.graph_c1;c45040.graph_c0
GO:0016310	Phosphorylation	3	159	c49263.graph_c1;c49263.graph_c0;c47972.graph_c0
GO:0006096	Glycolysis	4	26	c49263.graph_c1;c57401.graph_c0;c41007.graph_c0;c49263.graph_c0
GO:0010998	regulation of translational initiation by eIF2 alpha phosphorylation	1	1	c63835.graph_c0
GO:0035076	ecdysone receptor-mediated signaling pathway	1	1	c45040.graph_c0
GO:0006002	fructose 6-phosphate metabolic process	2	2	c49263.graph_c1;c49263.graph_c0
GO:0010506	regulation of autophagy	1	6	c63835.graph_c0
GO:0055114	oxidation-reduction process	1	223	c57401.graph_c0
GO:0006979	response to oxidative stress	1	20	c20754.graph_c0
GO:0009408	response to heat	1	15	c20754.graph_c0
GO:0055085	transmembrane transport	1	81	c52669.graph_c0
GO:0016311	dephosphorylation	1	27	c25738.graph_c0
GO:0043401	steroid hormone mediated signaling pathway	1	16	c45040.graph_c0
GO:0008340	determination of adult lifespan	1	54	c20754.graph_c0
**Molecular_function**				
GO:0004721	phosphoprotein phosphatase activity	1	47	c25738.graph_c0
GO:0016787	hydrolase activity	2	356	c55187.graph_c0;c41007.graph_c0
GO:0050661	NADP binding	1	9	c57401.graph_c0
GO:0004365	glyceraldehyde-3-phosphate dehydrogenase (NAD+) (phosphorylating) activity	1	2	c57401.graph_c0
GO:0004634	phosphopyruvate hydratase activity	1	2	c41007.graph_c0
GO:0022857	transmembrane transporter activity	1	21	c52669.graph_c0
GO:0000978	RNA polymerase II core promoter proximal region sequence-specific DNA binding	1	3	c49393.graph_c1
GO:0003872	6-phosphofructokinase activity	2	2	c49263.graph_c1;c49263.graph_c0
GO:0030246	carbohydrate binding	1	20	c51311.graph_c0
GO:0005496	steroid binding	1	1	c45040.graph_c0
GO:0004054	arginine kinase activity	1	2	c47972.graph_c0
GO:0000287	magnesium ion binding	1	22	c41007.graph_c0
GO:0004884	ecdysteroid hormone receptor activity	1	1	c45040.graph_c0
GO:0003756	protein disulfide isomerase activity	1	5	c38096.graph_c1
GO:0042302	structural constituent of cuticle	2	52	c53503.graph_c0;c39692.graph_c0
GO:0000982	RNA polymerase II core promoter proximal region sequence-specific DNA binding transcription factor activity	1	1	c49393.graph_c1
GO:0051287	NAD binding	1	18	c57401.graph_c0
GO:0043565	sequence-specific DNA binding	1	107	c45040.graph_c0
GO:0008184	glycogen phosphorylase activity	1	1	c46569.graph_c0
GO:0008270	zinc ion binding	1	214	c45040.graph_c0
GO:0005509	calcium ion binding	1	132	c27508.graph_c0
GO:0005488	binding	1	451	c48138.graph_c0
GO:0005524	ATP binding	3	563	c49263.graph_c0;c49263.graph_c1;c47972.graph_c0
GO:0030170	pyridoxal phosphate binding	1	30	c46569.graph_c0
**Cellular_component**				
GO:0005634	nucleus	2	429	c45040.graph_c0;c49393.graph_c1
GO:0005886	plasma membrane	1	132	c38096.graph_c1
GO:0005737	cytoplasm	1	267	c57401.graph_c0
GO:0005945	6-phosphofructokinase complex	2	2	c49263.graph_c1;c49263.graph_c0
GO:0000015	phosphopyruvate hydratase complex	1	2	c41007.graph_c0
GO:0016021	integral component of membrane	1	383	c52669.graph_c0
GO:0005740	mitochondrial envelope	1	4	c52669.graph_c0

**Fig 9:**
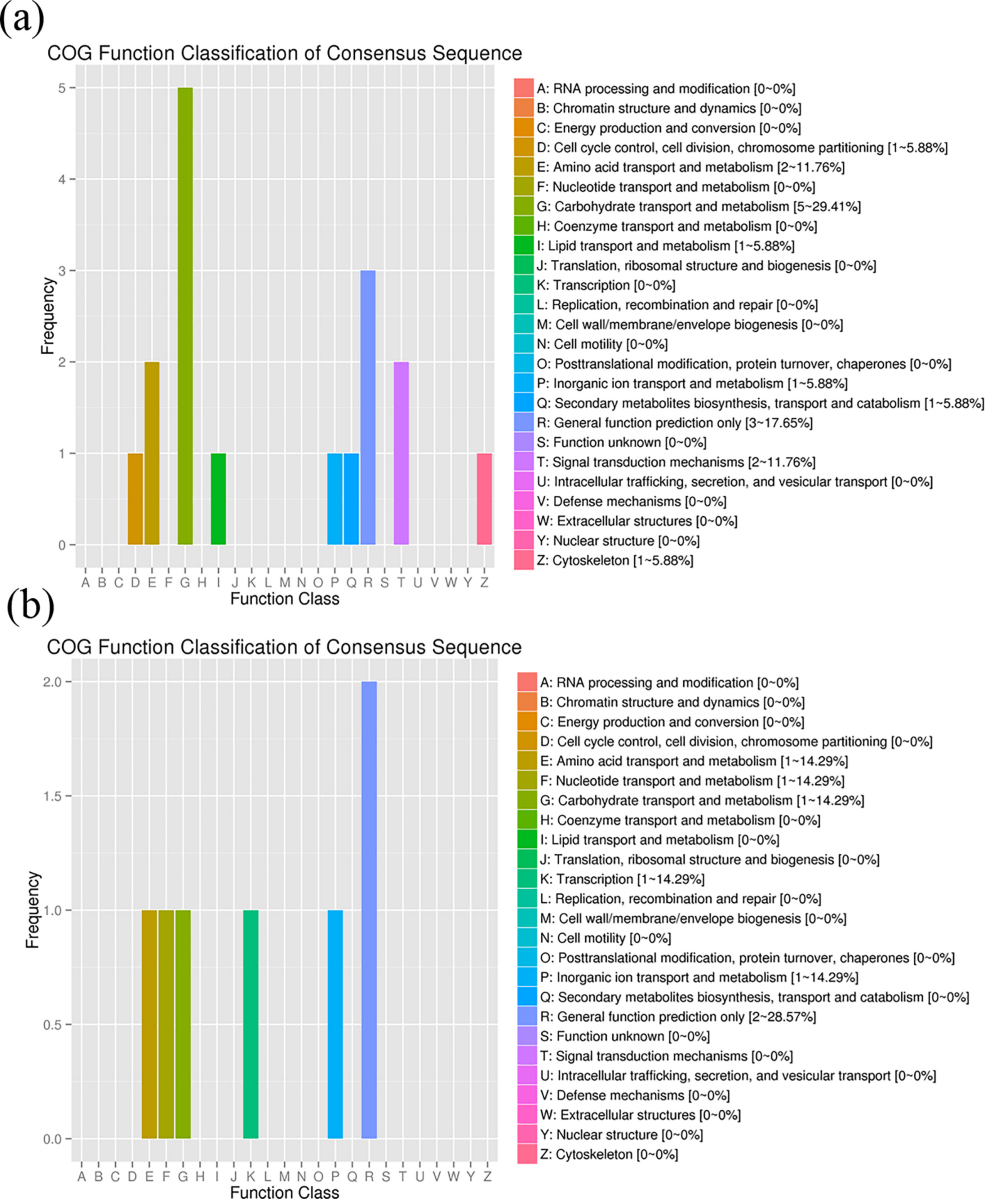
The COG categories of the DEGs of muscle. (a) The COG categories of the DEGs in stage D specimens and stage C specimens. The x-axis indicated COG process; the y-axis represented the number of the DEGs; (b) The COG categories of the DEGs of muscle in stage B specimens and stage C specimens.

COG function classification was performed with significant DEGs. In the comparison of stage D and stage C, a total number of 17 (17.2%) genes were COG-categorized into nine COG domains, 5 genes of which were in “carbohydrate transport and metabolism”, followed by 3 genes in “general function prediction only”, 2 genes in “signal transduction mechanisms” and 2 genes in “amino acid transport and metabolism”, one gene in the remaining domains (Fig 9A). In the comparison of stage B and stage C, 2 genes were found in “general function prediction only”, and 1 gene was in the rest domains, including “Transcription”, “Carbohydrate transport and metabolism”, “Amino acid transport and metabolism”, “Nucleotide transport and metabolism” and “Lipid transport and metabolism” (Fig 9B).

**Fig 10:**
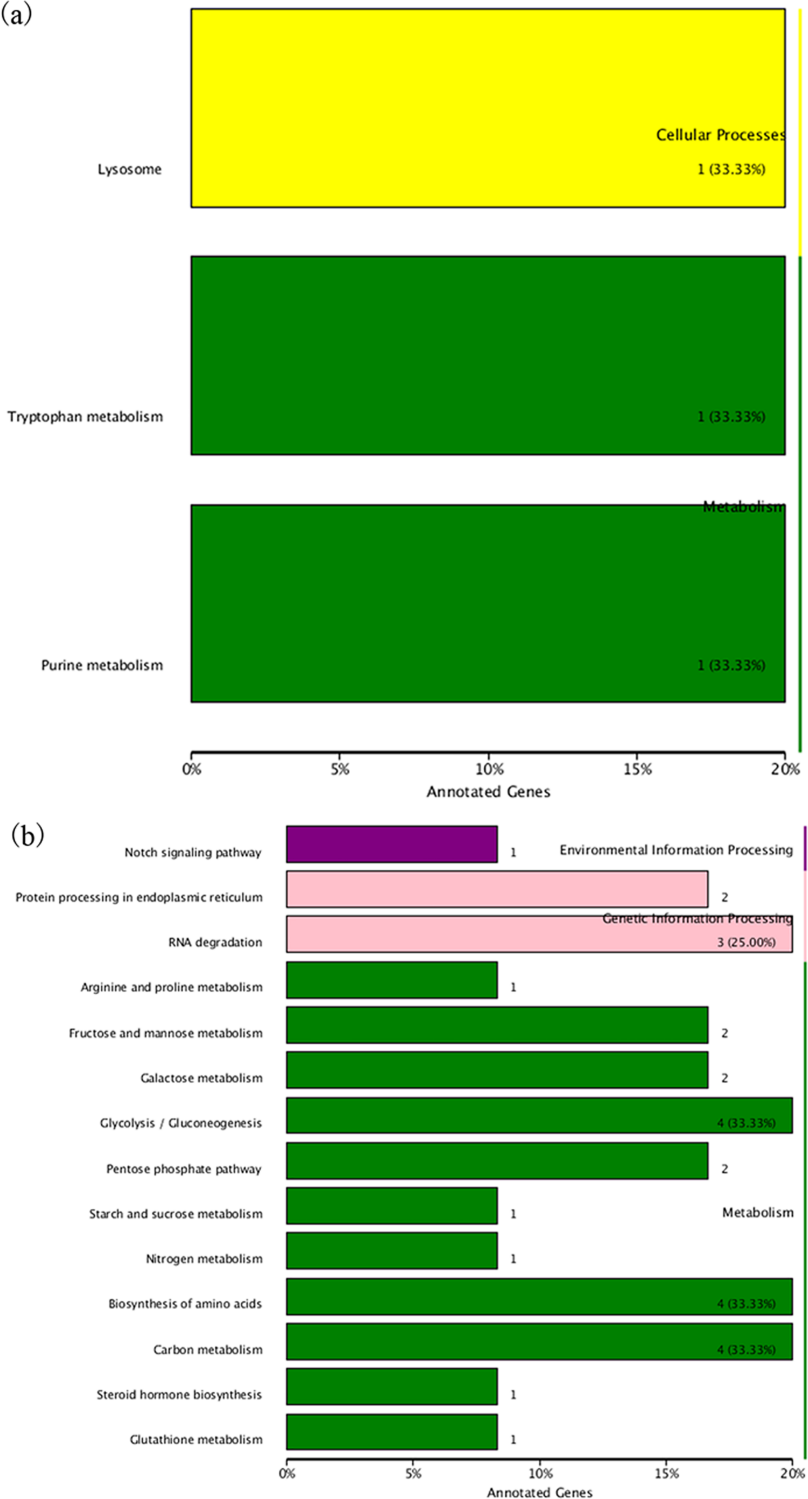
The KEGG pathways of the DEGs of muscle. (a) The KEGG pathways of the DEGs in stage C specimens and stage B specimens. The x-axis indicated the proportion of annotated DEGs in the pathway. The numbers of genes in the pathway were recorded. The y-axis represented the pathway. The same color-coded pathway was in the same KEGG category; (b) The KEGG pathways of the DEGs of muscle in stage C specimens and stage D specimens.

In comparison of stage B and stage C, 3 genes were classified into cellular processes and metabolism. The genes were assigned to transport and catabolism related to lysosome (ko: 04142), amino acid and nucleotide metabolism related to purine metabolism (ko: 00230) and tryptophan metabolism (ko: 00380), respectively (Fig 10A). In comparison of stage D and stage C, 19 genes were classified into environmental information processing, genetic information processing and metabolism (Fig 10B). In environmental information processing, one gene related to signal transduction was found in Notch signaling pathway (ko: 04330); five folding, sorting and degradation related genes were found in genetic information processing, two of which were assigned to protein processing in endoplasmic reticulum (ko: 04141) and the other were assigned to RNA degradation (ko: 03018). Glycolysis/gluconeogenesis, carbon metabolism and biosynthesis of amino acids were the top three pathway in metabolism (23 genes), with four genes respectively.

## Discussion

A total of 127.87 Gb clean data consisting of 94,853 unigenes were successfully obtained from the mud crab *S*. *paramamosain* at three fattening stages. Previous study has shown 21,791 isotigs in the testis and ovary of the same crab species [8]. In addition, the gills of this crab species challenged with mud crab reovirus was analyzed, producing 67,279 assembled unigenes [9]. The unigenes identified in the study not only enrich transcriptomes data of *S*. *paramamosain*, but also provide the basis for studying the mechanisms of muscle growth.

In this study, 23,787 genes could be annotated. Main gene categories were found to be involved in ATP binding and catalytic activities, metabolic and oxidation-reduction processes and nucleus and membrane, which are necessary to maintain the basic life activities. The genes in these categories are relatively conservative [22, 23]. The annotation rate was lower than previous reports in yesso scallop *Patinopecten yessoensis* and sea cucumber *Apostichopus japonicus*, whose annotation rate was 27.9% and 39.1%, respectively [24, 25]. The low rate of annotated gene might be due to insufficient of the genomic data of *S*. *paramamosain*. The potentially available and unexploited genes, especially DEGs might play an important role in the process of muscle growth.

SNPs were used in trait-mapping and whole-genome association studies owing to the variability and abundance in the genome [4, 26]. SNPs, as potential markers, were even found in the specie without the full genome sequences [27, 28]. In the current study, 451,564 SNPs were called using GATK, with approximately 34-folds increase in comparison with the 13,271 SNPs in the gonads [8]. The putative SNPs could be useful in various fields of aquaculture of *S*. *paramamosain*, for example, conservation and population genetics of wild species, mapping of economically important traits, selection and breeding programmes [29]. In addition, a total of 177 SNPs were found in 31 DEGs, including 105 SNPs in 12 annotated genes. Previous study has described that SNPs in *actin* and *CHH* could affect the growth in the giant freshwater prawn *Macrobrachium rosenbergii* [30]. Therefore, SNPs in DEGs are speculated to affect the muscle growth during fattening process.

In crustaceans, the muscle growth and development is very complicated, and many details remain unknown to date. Muscle growth is accompanied with the increase in the number and length of muscle fibers [5]. There is few definite report on increasing the number of muscle fibers. But, once specific muscles have been established during the early stages of development, muscles continue to grow in both length and diameter [31]. In fully differentiated lobster, the muscle fibers grow in length by the addition of sarcomeres [6], and grow in cross-sectional area by the splitting of large myofibrils upon molting and enlargement during the inter-molt [6]. In crayfish *Procambarus clarkii*, increase in fiber length is accomplished by lengthening of existing sarcomeres [32]. Previous study speculated that the muscle growth could be stimulated by fibers stretching due to the expansion of the new exoskeleton during the post-molt period [33]. However, the mechanism involved in muscle growth is unclear in the mud crab *S*. *paramamosain*.

Extensive studies show that muscle growth responds to exercise, nutrient supply, metabolism, cytokines and endocrine factors and denervation [34]. In *Drosophila*, muscle growth is associated with nutrient sensing by the Insulin/Akt/TOR pathway, which was achieved by the integrated regulation of the transcription factors FOXO, Myc and Mnt [34–36]. The biosynthesis promotes formation of syncytial muscle, which can be stimulated by glycolysis [36]. In addition, glycolysis/pyruvate metabolism has a negative effect on the Notch pathway, and which were regulated by insulin pathway or target of rapamycin pathways [37]. Tixier et al. reported that seven genes (*Pglym78*, *Pfk*, *Tpi*, *Gapdh*, *Pgk*, *Pyk*, and *Impl3*) involved in glycolysis/pyruvate metabolism, play a vital role in the increasing of muscle fibers size and the myoblast fusion [36]. Some conserved cytokines could activate the pathways associated with muscle growth. For example, *Drosophila eiger* (*CG12919*), *outstretched*, *unpaired 2*, and *unpaired 3*, which regulate the muscle development by activation of the TNF signaling cascade and the Eya and JAK-STAT signaling [38, 39]. Octopamine receptor 2 causes muscle hypertrophy by regulating synthesis of cyclic-AMP and PKA [40]. The study on the interconnections between exercise and muscle mass highlighted that spargel promotes mitochondrial activity and is required for sensing the physiological effects caused by exercise [34, 41]. The muscle-nerve interactions play an important role in the formation of muscle size and structure in *Drosophila* [42]. In addition, a lot of factors were proven to be involved in the atrophy of muscle, such as *Drosophila* (*CG11658*, *abba*, *CG10961*, *Cbl*, *TER94 and CG6233*), p97/VCP ATPase complex, MLCs, actin and so on [43–46].

In this study, the claw muscle transcriptomes were sequenced for screening the functional genes that affect the muscle growth of *S*. *paramamosain*. The predominant catalogs of DEGs clusters were regulation of transcription, ATP binding and integral component of membrane. The results suggested that abundant DEGs involved in maintaining the basic life activities during different fattening stages. The enzymes, related to glycogen metabolism (enolase, glycogen phosphorylase) and protein metabolism (arginine kinase, aminopeptidase N), were up-regulated at stage C specimens compared to stage D specimens, which may take part in maintaining metabolic balance during fattening process. Furthermore, several DEGs have been found, which play roles in skeletal muscle differentiation, atrophy and transformation in *Drosophila* or mice, such as *Pfk-1*, *Gapdh*, *Hspb6*, *Su (H)*, *Sspn* and *Mlc* [36, 46–49].

PFK-1 and GAPDH are the basic enzymes in glycolysis. Previous study showed that PFK-1 is regulated by signals from cell proliferation [50, 51]. In addition, PFK-1 regulates the muscle fatigue by interaction with neuronal nitric oxide synthase [52]. PFK-1 and GAPDH play important roles in the increasing of muscle fibers size and the myoblast fusion in *Drosophila* [36]. In this study, they were found in claw muscle transcriptomes and up-regulated at stage C specimens compared to stage D specimens. The results suggest that they could participate in the muscle growth. As it is known that the muscle fibers rely mainly on glycolysis [53], the high expression of PFK-1 and GAPDH also may be associated with higher energy metabolism at stage C specimens.In *D*. *melanogaster*, the notch signalling pathway can regulate the differentiation of muscle [54]. Su (H) functions as a transcriptional repressor, has been shown to participate in the notch signaling pathway and the myogenesis [46]. Myogenesis in crustaceans may share some similarities with *Drosophila*. Su (H) was found with higher expression in stage C_3/4_ specimens than in stage D_3_ specimens in muscle transcriptomic database, and it is likely to be involved in the myogenesis.

HSPB6, as a member of the small heat shock protein family, was constitutively expressed in skeletal muscle [55]. HSPB6 not only plays a role in prevention of atrophy, ischemia, hypertensive stress, and metabolic dysfunction, but also regulate muscle contraction by binding to the troponin complex [47, 56]. In the present study, twelve HSPB6 transcripts were up-regulated expression in claw muscle under stage C_3/4_ specimens compared to the stage D_3_ specimens. The claw muscular atrophy occurred mainly at the stage D_3_. The high expression of these genes suggest that HSPB6 could be involved in the muscle protection against atrophy by stabilizing myofibrillar proteins at stage C_3/4_.

In mice, Sarcospan (SSPN) plays important roles in muscular dystrophy and muscle force development by linked to sarcoglycans or as a component of the dystrophin-glycoprotein complex [48, 57, 58, 59]. SSPN was discovered in the claw muscle transcriptome that has not been identified previously in *S*. *paramamosain*. According to the function of SSPN reported in mice, SSPN may be involved in muscle atrophy of *S*. *paramamosain*. MLCs, as the myofibrillar isoform, expressed to varying degrees in different fiber types in crustacean muscle. And the contractile apparatus transformation is accompanied by change in fiber types, which can significantly affect muscle size [49]. In addition, MLCs has been found to play a key role in atrophy, which can regulate the extraction effect of ubiquitinated proteins from the myofibrils [44]. In this study, MLCs expression may be consistent with the contractile apparatus transformation.

In addition, 34 neuropeptides were found in the transcriptome data of claw muscle in *S*. *paramamosain*. Some of the neuropeptides (e.g., the AST-B, sNPF, NP, CHH and orcokinin, DH31) had been confirmed to be widely expressed in various tissues (e.g., the cerebral ganglia and ovary) of this crab species [7]. Neuropeptides may be secreted by neuroendocrine in the nervous system and autocrines/paracrines in the non-nervous system, which could regulate many physiological processes, including growth, locomotion, reproduction and metabolism [7, 60, 61]. The neuropeptides in claw muscle might function as neurotransmitters or neuromodulators to regulate muscle growth.

## Conclusions

The first transcriptome analysis on the claw muscle of *S*. *paramamosain* was carried out successfully and yielded 35.46 Gb clean data. Data obtained in present study greatly contributes to the understanding of the gene expression and genome structure occurring within the claw muscle of *S*. *paramamosain* at different molting stages. Potential SNPs found in the transcriptome are useful for future selective breeding, trait-mapping, and gene localization studies. The discovery and validation of DEGs showed that these particular genes might be conducive to claw muscle atrophy or restoration in *S*. *paramamosain*.

## Supporting information

S1 Table Sequences of unigenes in *S*. *paramamosain*. (ZIP)

S2 Table Unigene annotation in *S*. *paramamosain*. (XLS)

S3 Table The SSRs identified in unigenes in *S*. *paramamosain*. (XLS)

S4 Table The DEGs of muscle in *S*. *paramamosain*. (XLS)

S5 Table The SNPs in DEGs of muscle in *S*. *paramamosain*. (XLSX)

S1 Fig The length distribution of cds in *S*. *paramamosain*. The x-axis indicated the length of cds; the y-axis indicated the number of cds.
(TIF)

S2 Fig Analysis of DEGs of muscle in the stage B specimens compared to the stage D specimens. The different color dots indicate the significant DEGs (p < 0.01). Red dots indicate the DEGs with log_2_fold change greater than 2 (up-regulated in post-molt specimens) and green dots indicate genes with log_2_fold change less than -2 (down-regulated in stage B specimens).
(TIF)

S3 Fig The DEGs of muscle in the stage B specimens compared to the stage C specimens. Red dots indicate the DEGs up-regulated in post-molt specimens and green dots indicate DEGs down-regulated.
(TIF)

S4 Fig The DEGs of muscle in the stage D specimens compared to the stage C specimens. Red dots indicate the DEGs up-regulated in stage D specimens and green dots indicate the DEGs down-regulated.
(TIF)
