# Supplementary figures and images for: Transcriptome profiling of claw muscle of the mud crab (Scylla paramamosain) at different fattening stages
Source: PLoS One. 2017 Nov 15;12(11):e0188067. doi: 10.1371/journal.pone.0188067 (PMC5687733; doi:10.1371/journal.pone.0188067)

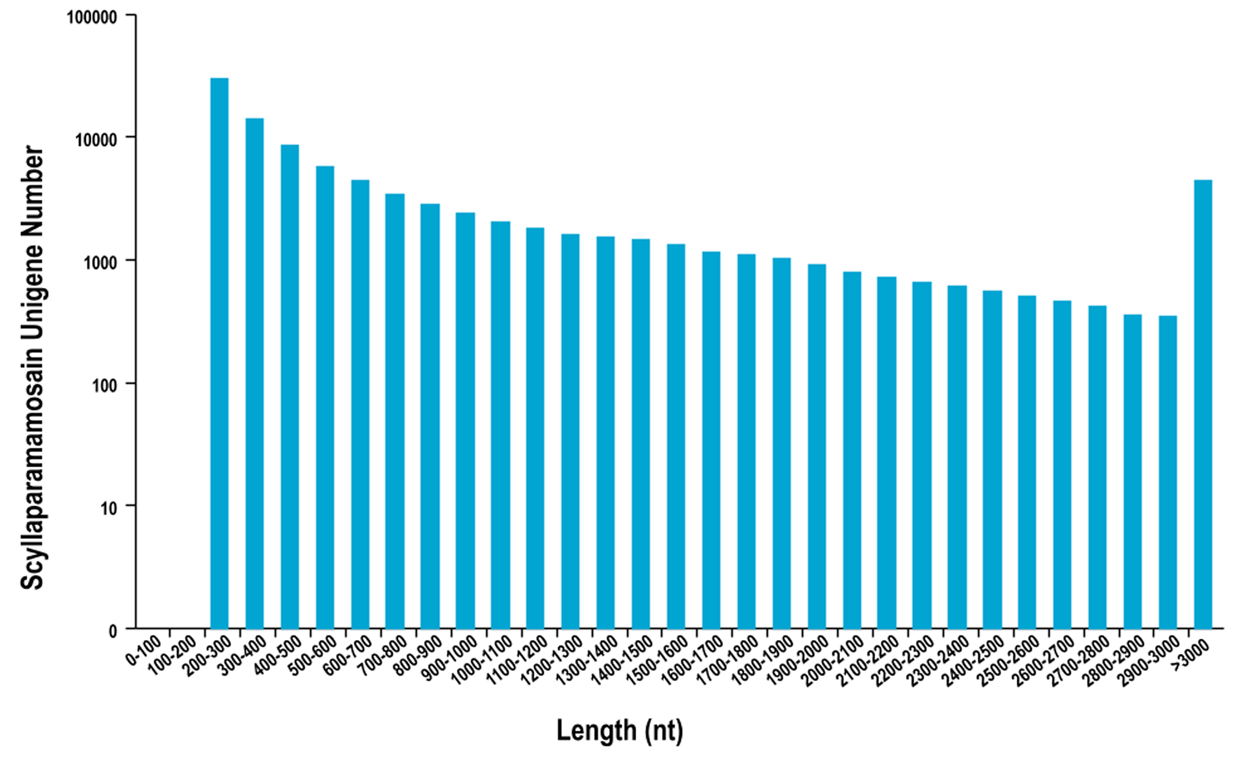

Supplement: S1 Fig — The x-axis indicated the length of cds; the y-axis indicated the number of cds. (TIF) [file pone.0188067.s006.tif]

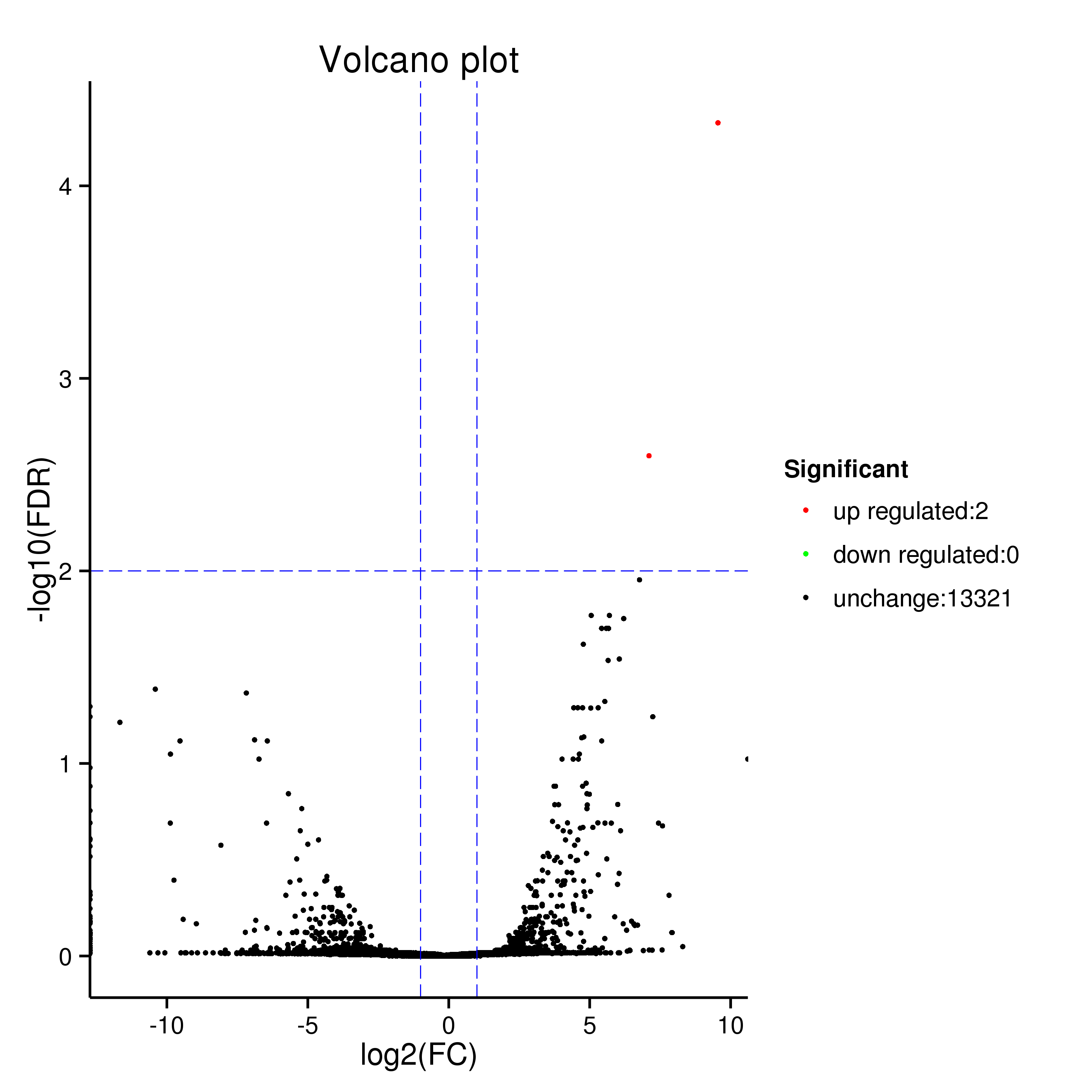

Supplement: S2 Fig — The different color dots indicate the significant DEGs (p < 0.01). Red dots indicate the DEGs with log2fold change greater than 2 (up-regulated in post-molt specimens) and green dots indicate genes with log2fold change less than -2 (down-regulated in stage B specimens). (TIF) [file pone.0188067.s007.tif]

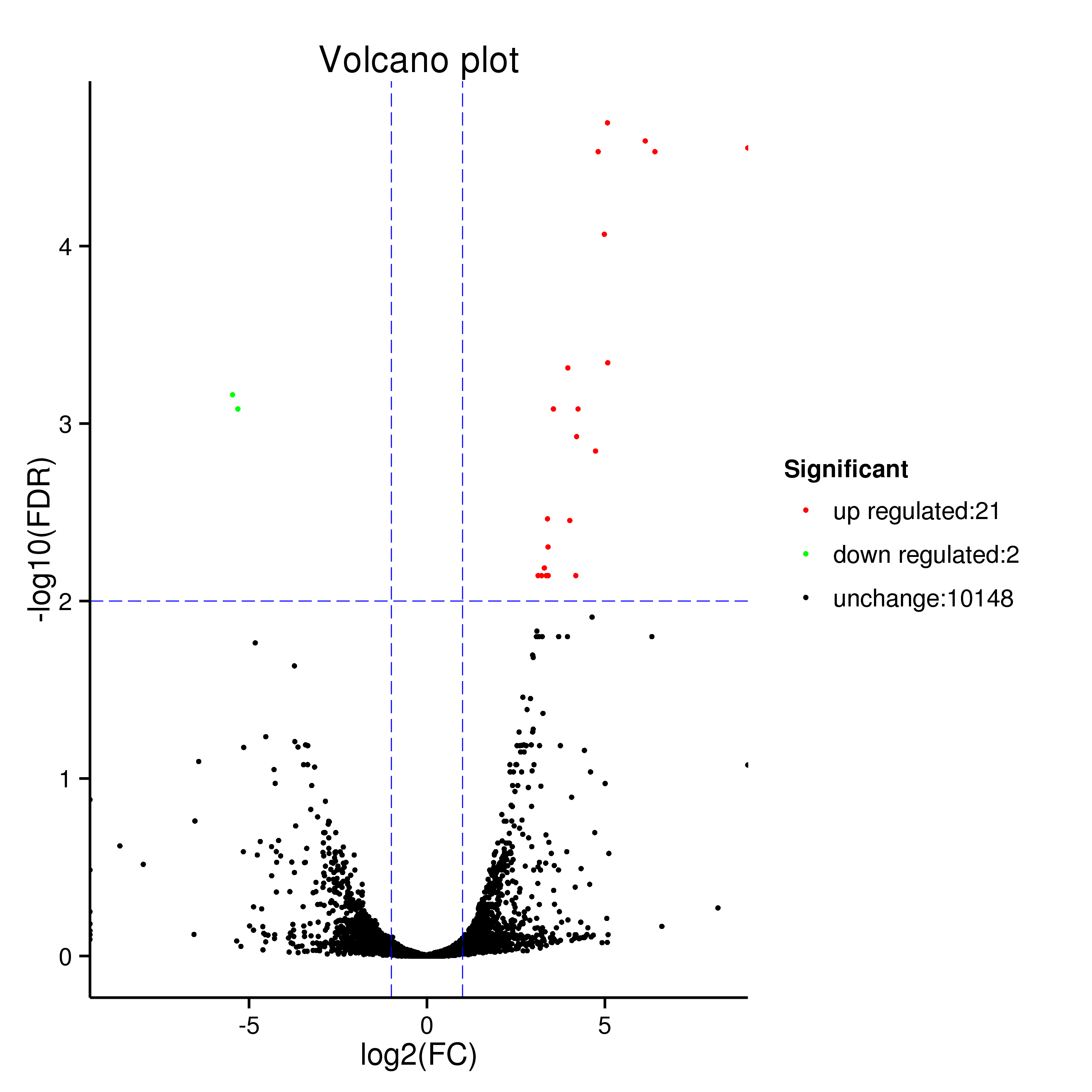

Supplement: S3 Fig — Red dots indicate the DEGs up-regulated in post-molt specimens and green dots indicate DEGs down-regulated. (TIF) [file pone.0188067.s008.tif]

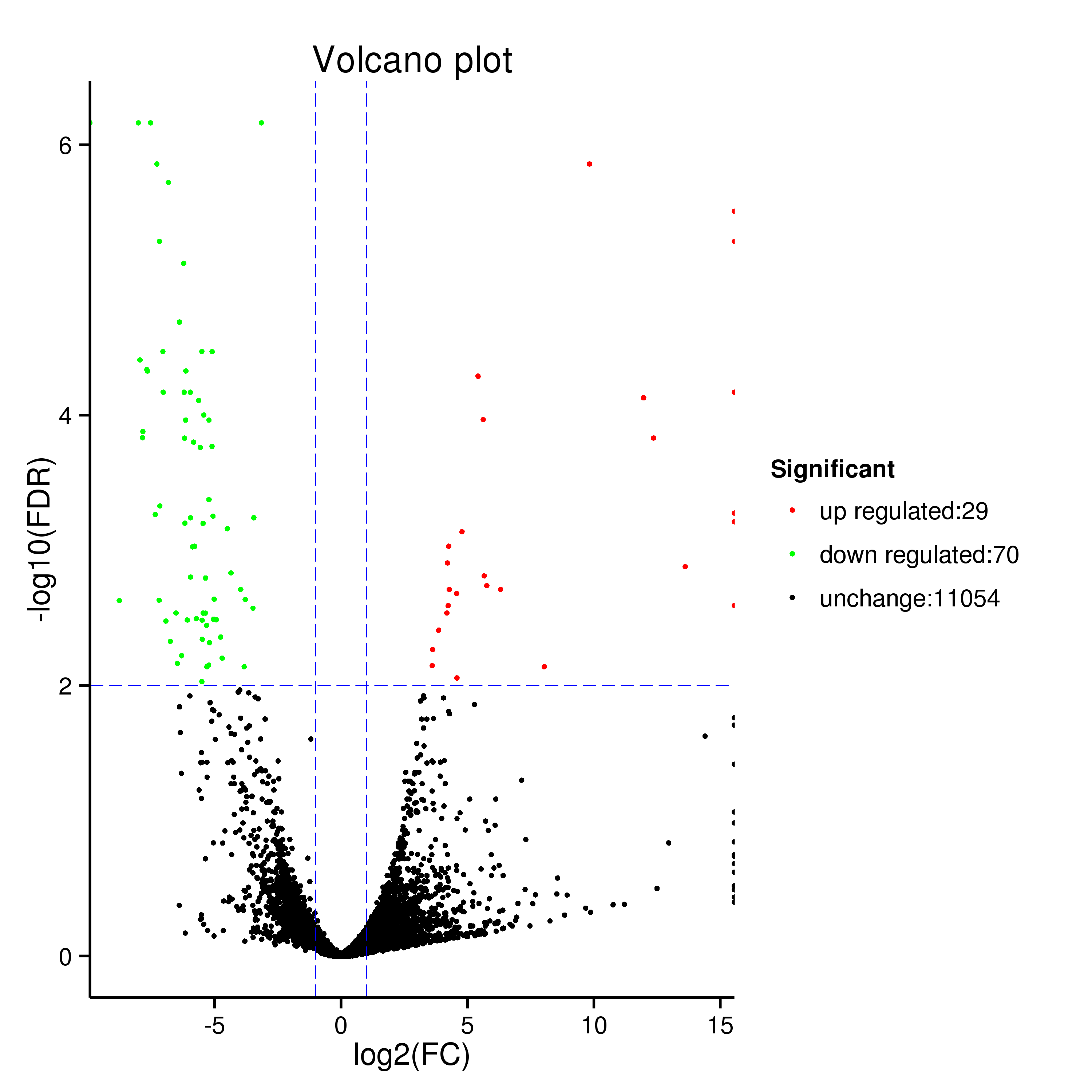

Supplement: S4 Fig — Red dots indicate the DEGs up-regulated in stage D specimens and green dots indicate the DEGs down-regulated. (TIF) [file pone.0188067.s009.tif]
